# Integrating Rapid Evaporative Ionization Mass Spectrometry Classification with Matrix-Assisted Laser Desorption Ionization Mass Spectrometry Imaging and Liquid Chromatography-Tandem Mass Spectrometry to Unveil Glioblastoma Overall Survival Prediction

**DOI:** 10.1021/acschemneuro.4c00463

**Published:** 2025-02-26

**Authors:** Tim F.E. Hendriks, Angeliki Birmpili, Steven de Vleeschouwer, Ron M.A. Heeren, Eva Cuypers

**Affiliations:** 1 The Maastricht MultiModal Molecular Imaging (M4I) Institute, Division of Imaging Mass Spectrometry (IMS), Maastricht University, Maastricht 6229 ER, The Netherlands; 2 Department of Neurosurgery, UZ Leuven, and Laboratory for Experimental Neurosurgery and Neuroanatomy, Department of Neurosciences and Leuven Brain Institute (LBI), KU Leuven, Leuven 3000, Belgium

**Keywords:** glioblastoma, survival time, REIMS, MALDI-MSI, proteomics

## Abstract

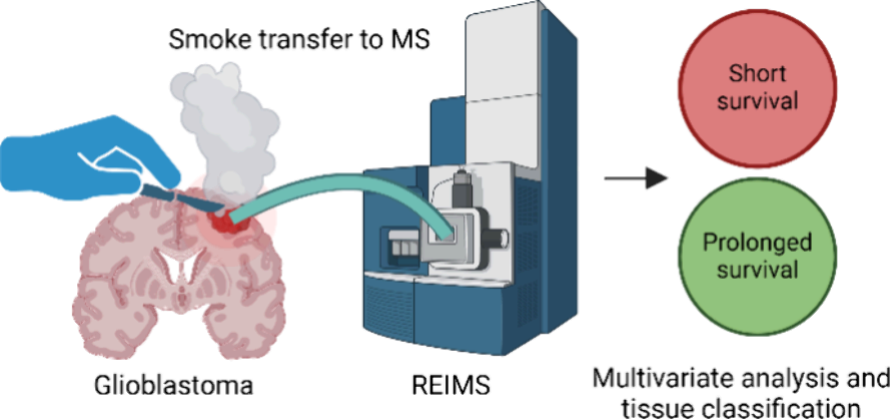

Glioblastoma multiforme (GBM) is a highly aggressive brain cancer with a median survival of 15 months. Despite advancements in conventional treatment approaches such as surgery and chemotherapy, the prognosis remains poor. This study investigates the use of rapid evaporative ionization mass spectrometry (REIMS) for real-time overall survival time classification of GBM samples and uses matrix-assisted laser desorption ionization mass spectrometry imaging (MALDI-MSI) to compare lipidomic differences within GBM tumors. A total of 45 GBM biopsies were analyzed to develop a survival prediction model for IDH-wild type GBM. REIMS patterns from 28 patients were classified with a 97.7% correct classification rate, identifying key discriminators between short-term (0–12 months) and prolonged (>12 months) survivors. Cross-validation with additional samples showed that the model correctly classified short-term and prolonged survival with 66.7 and 69.4% accuracy, respectively. MALDI-MSI was performed to confirm the discriminators derived from REIMS data. Results indicated 42 and 33 discriminating features for short-term and prolonged survival, respectively. Proteomic profiling was performed by isolating tumor regions via laser-capture microdissection (LMD) and liquid chromatography-tandem mass spectrometry (LC-MS/MS). Subsequently, 1387 proteins were identified, of which 79 were significantly altered. In conclusion, this study shows that REIMS rapidly predicts glioblastoma survival times based on lipidomic profiles during electrosurgical dissection. MALDI-MSI confirmed that these differences were specific to the tumor region in the glioblastoma sections. LMD-guided LC-MS/MS-based proteomics revealed significantly altered pathways between short-term and prolonged survival. This research, including the comprehensive predictive survival model for GBM, could guide tumor resection surgeries based on accurate real-time tumor tissue identification as well as provide insights into overall survival mechanisms, possibly related to therapy response.

## Introduction

Glioblastoma multiforme (GBM) stands as the most aggressive and lethal form of brain cancer, characterized by its rapid progression and median survival prognosis of 15 months after standard-of-care therapy.^1,2^ Among all the traditional treatment methods including chemotherapy and radiation, the first-line treatment of GBM is surgery.^3−6^ Here, as much of the tumor tissue is removed with as few impairments as possible to the surrounding brain tissue still responsible for normal neurological function.^7^ Even with advancements in treatment modalities, the median survival for patients diagnosed with GBM remains poor, underscoring the urgent need for innovative diagnostic and prognostic tools.^8^ Regarding the diagnosis, conventional radiologic techniques such as a computed tomography (CT) scan and magnetic resonance imaging (MRI) provide valuable information about tumor size and grade.^9−11^ However, histological and molecular pathological examination is currently considered the gold standard in GBM diagnosis.^12^ The entire procedure is labor-intensive, time-consuming, and subjective to interpretation.^13,14^ Thus, the current histopathology diagnostic method is slow and can delay the postoperative treatment plan of the patient.

Studies indicate that the conventional central nervous system (CNS) tumor examination, based on WHO guidelines, could not describe the biological characteristics of gliomas and lacked the capability to estimate the clinical outcome of gliomas.^15^ The WHO classification of CNS tumors is defined by both histology and molecular features such as isocitrate dehydrogenase 1 (IDH)-wild type (IDH^WT^) and IDH mutant (IDH^M^) variants.^15^ These subtypes are characterized by the presence or absence of mutations in the IDH gene. Conventionally, to better predict the survival time for glioma patients, emphasis is put on the identification of these molecular features.^16,17^ IDH^M^ glioma patients typically survive longer than IDH^WT^ patients.^18^ Increasing the survival time by improving the treatment approach of brain tumor patients has been a focus of many healthcare specialists.^19^ The success of the treatment depends on several factors such as the extent of resection, sensitivity of the tumor to chemoradiation therapy, and the molecular characteristics of the tumor.^18,20^

Mass spectrometry (MS) is a powerful analytical technique renowned for its capability to measure molecules with high sensitivity, specificity, speed, and accuracy. Its versatile applications span across various domains including lipidomics, proteomics, and metabolomics, therapeutic drug monitoring (TDM), and disease diagnosis within clinical research.^21−24^ Rapid evaporative ionization mass spectrometry (REIMS) is an emerging technology based on MS analysis of aerosols generated during thermal ablation of biological samples. REIMS is capable of quasi-instantaneous real-time classification of samples based on molecular profiling using a predetermined molecular library.^25,26^ Real-time tissue classification is especially useful in the context of cancer surgery where the objective is to gain rapid information about the tumor characteristics but also the location and extent of the tumor. REIMS has already been successfully tested in glioblastoma margin delineation and evaluating the extent of cancer cell infiltration.^27^ The molecular characteristics of the tumor determine the next step in the surgery and are crucial for patient outcomes.^27,28^

Surgical resection often provides limited information due to high spatial heterogeneity.^29^ Matrix-assisted laser desorption ionization mass spectrometry imaging (MALDI-MSI) enables spatially resolved molecular profiling of tissue sections, allowing for the visualization and identification of biomolecules within the heterogeneous tumor microenvironment.^30^ MSI in its many forms provides insights into the heterogeneity and metabolic dynamics of GBM, which are key to understanding disease progression, treatment response, and outcomes.^31,32^ Additionally, liquid chromatography-tandem mass spectrometry (LC-MS/MS) proteomics offers a complementary approach for increasing the understanding of the lipidomic and proteomic landscape in GBM. As previously described, LC-MS/MS enables the identification of lipids and proteins associated with tumor aggressiveness and in turn patient survival.^33,34^

In this study, a survival prediction model for IDH^WT^ human GBM based on REIMS spectra is shown. Furthermore, by using MALDI-MSI, the lipidomic differences between short-term (0–12 months) and prolonged (>12 months) survival are visualized and are compared with the REIMS spectra based within the GBM tumor. To identify the lipids in the tumor region, a single-section approach for lipidomics as previously described was used.^35^ The hypothesis is that differences in survival time within GBM patients are determined by differences within the lipidomic profile. These differences can be quasi-instantaneously measured via REIMS, and survival time can be predicted via a principal component analysis-linear discriminant analysis (PCA/LDA) model based on a spectral library. Afterward, via the acquired proteomic data, the results are correlated with known biomarkers and pathways. By integrating the spatial distribution of lipids within GBM tissues using MSI and LC-MS/MS, coupled with the rapid and high-throughput capabilities of REIMS, we aim to develop a comprehensive predictive survival model that combines lipidomic and proteomic data. Our goal is to enhance the accuracy and clinical utility of overall survival predictions, thereby facilitating personalized treatment strategies and improving patient outcomes.

## Results

### Patient and Clinical Characteristics

A prospective cohort of 45 GBM samples was analyzed after excluding any IDH^M^ tumors (Supplementary Table S1). Thirty-one patients (69%) were male and 14 (31%) were female with the median age at diagnosis being 60 (interquartile range (IQR), 51–69). The tumor location varied with half of the cases found either at the frontal or parietal lobe and the rest at the temporal lobe. The glial fibrillary acidic protein (GFAP) and α-thalassemia X-linked intellectual disability (ATR-X) syndrome were noted in 32 patients, coexisting in 20 of them. Nineteen of the sample population showed mutations in the p53 tumor suppressor gene, six had high expression of Ki67, and there was one case with a 1p/19q codeletion. Among the mentioned markers, the presence of ATR-X syndrome, p53 mutations, and high Ki67 expression is associated with a worse prognosis in glioblastoma patients.^36−38^ In contrast, the 1p/19q codeletion is linked to a better response to therapy and a better prognosis^37^ and typically required for IDH^M^ oligodendrogliomas. GFAP is a marker for astrocytic cells, and its expression is typically used to confirm the diagnosis of glioblastoma rather than provide prognostic information.^39^ Nevertheless, there were no consistent patterns identified between the overall survival of the patients involved in the study and the genetic mutations noted. A radiochemotherapy treatment with temozolomide (TMZ) of up to 9 cycles was initiated in 40 patients (83%), 2 were administrated lomustine (1) and dabrafenib (1) since TMZ admission was observed to be ineffective, and supportive care was provided to three patients due to their decision (no treatment). There was no observed correlation between a specific type of chemotherapy and overall survival for the patients included in the study. A total of 80% of the patients died within the two-year range after diagnosis, while the median overall survival of the selected patients was 17.4 months.

### Overall Survival Model for Human Glioblastoma Using REIMS

**Figure 1:**
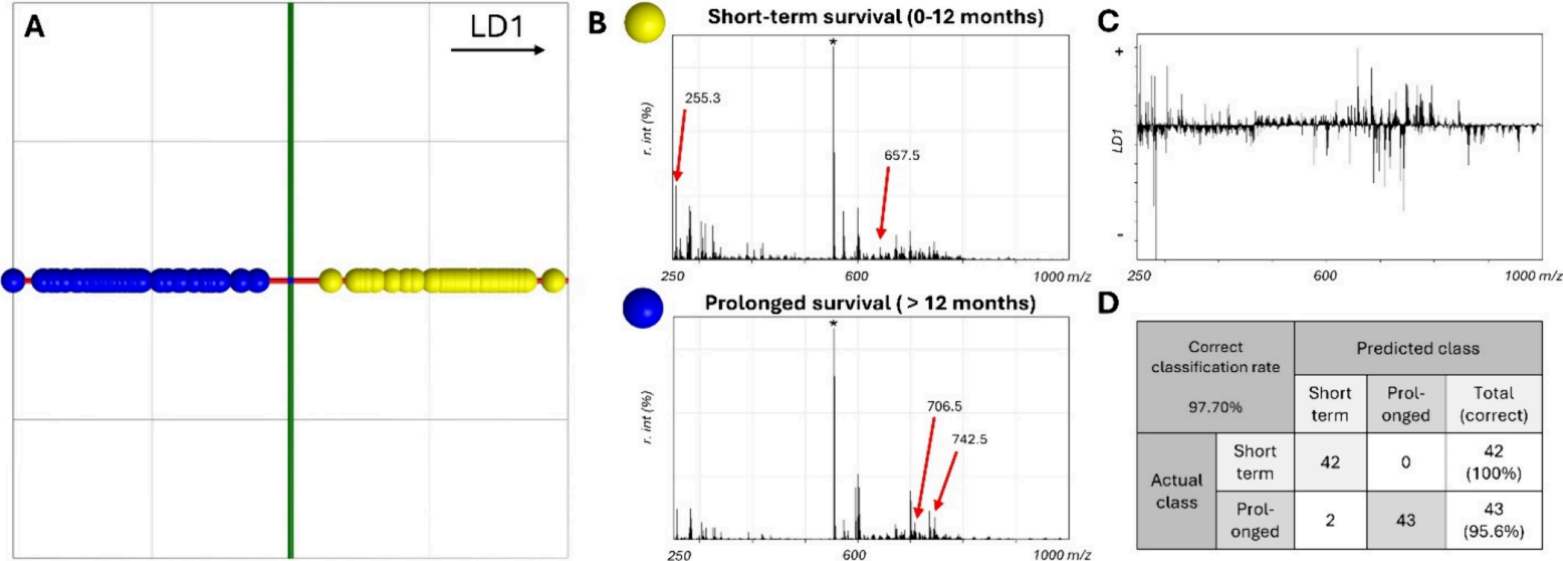
(A) LDA score plot at *m*/*z* 250–1000. OS 0–12 months (yellow) and OS > 12 months (blue) show clear separation in this LDA model. (B) Average mass spectra of the measurements used for their respective classes in the LDA model. The asterisk (*) indicates the Leu-Enk peak at *m*/*z* 554.25. (C) Mass features for loading discriminant (LD) 1. LD1 discriminates between (+) short-term and (−) prolonged survival. (D) Confusion matrix showing the overall correct classification rate and the correct classification rate per survival class.

The survival model was built based on 87 REIMS-generated patterns derived from 28 patients. That is because multiple incisions were made per biopsy to address tissue heterogeneity. Ultimately, 42 burns originated from samples associated with short-term survival (0–12 months) and 45 burns with prolonged survival (>12 months). Leucine-enkephalin, an endogenous peptide neurotransmitter that is naturally found in most tissue types in the human brain, was used as an internal standard prior to and during the measurements. We referred to the software and literature guidelines to decide on the optimal number of PCA components for our analysis, typically suggesting dividing the number of patterns by four.^40^ Based on this range, several models were developed with varying component numbers, manually testing their accuracy, and comparing the results with internal cross-validation results. The final model was selected based on the best alignment between their performances. A line chart displaying the accuracy trends for different component numbers has been included in the Supporting Information for further clarity (Supplementary Figure S1). An internal 20%-out cross-validation was conducted using the software employed for the model, following PCA/LDA feature extraction. REIMS patterns were classified with an overall accuracy of 97.70% (Figure 1a–d). The LDA score plot (Figure 1a) showed separation over the LD1 axis. Single mass patterns for short-term and prolonged survival (Figure 1b) reveal distinct differences between the groups, such as *m*/*z* 255.3 and 657.5 being prominent in short-term survival, while *m*/*z* 706.5 and 742.5 are more prevalent in prolonged survival. The LD1 score plot (Figure 1c and Supplementary Table S2) further highlights these *m*/*z* values as key discriminators, as their distance from the baseline reflects their contribution to the linear discriminant. There were certain masses observed in the lower end of the spectrum to be most abundant in the short-term patients, such as *m*/*z* 281.3 and *m*/*z* 255.3, assigned as oleic acid FA (18:1)^−H^ and palmitic acid FA (16:0)^−H^, respectively. Accordingly in the prolonged survivors, *m*/*z* 279.3 and *m*/*z* 283.3, assigned as linoleic acid FA (18:2)^−H^ and stearic acid FA (18:0)^−H^, were more prevalent. Nevertheless, differences between short-term and prolonged survival were also found within the lipid mass range. Masses *m*/*z* 657.5, *m*/*z* 682.5, *m*/*z* 747.6, and *m*/*z* 794.5 were considered to separate short-term survivors from the rest, while *m*/*z* 687.5, *m*/*z* 697.5, *m*/*z* 706.5, *m*/*z* 742.5, and *m*/*z* 862.7 mostly corresponded to the molecular profile of the prolonged survivors. The PCA visualization plot (Supplementary Figure S2) illustrates the distribution of the burning points in the reduced dimensional space and already shows some distinct clustering of the samples based on their overall survival, aligning with the LDA results. This supports the robustness of the classification model by highlighting the inherent patterns corresponding to different survival outcomes.

Additionally, the GBM survival model created with REIMS was validated by measuring 6 short-term and 11 prolonged survival GBM samples that were not previously included in the model. Supplementary Table S3 shows the classification given by the model of the burns generated from the unknown samples. When measuring an unidentified sample, the model is able to distinguish short-term and prolonged survival with 66.7 and 69.4% correct classification, respectively, based on the correctly classified burns. When considering correct classifications at the patient level, based on the majority of identified burns, the accuracy for short-term survival remained at 66.7%, while the accuracy for long-term survival decreased to 63.6%. These results indicate that the model demonstrates a promising level of accuracy, even when analyzing unknown patient samples. This further indicates the potential of the REIMS classification model as a valuable tool in predicting the survival time of a GBM patient.

### MALDI-MSI of Short-Term and Prolonged Survival Samples

**Figure 2:**
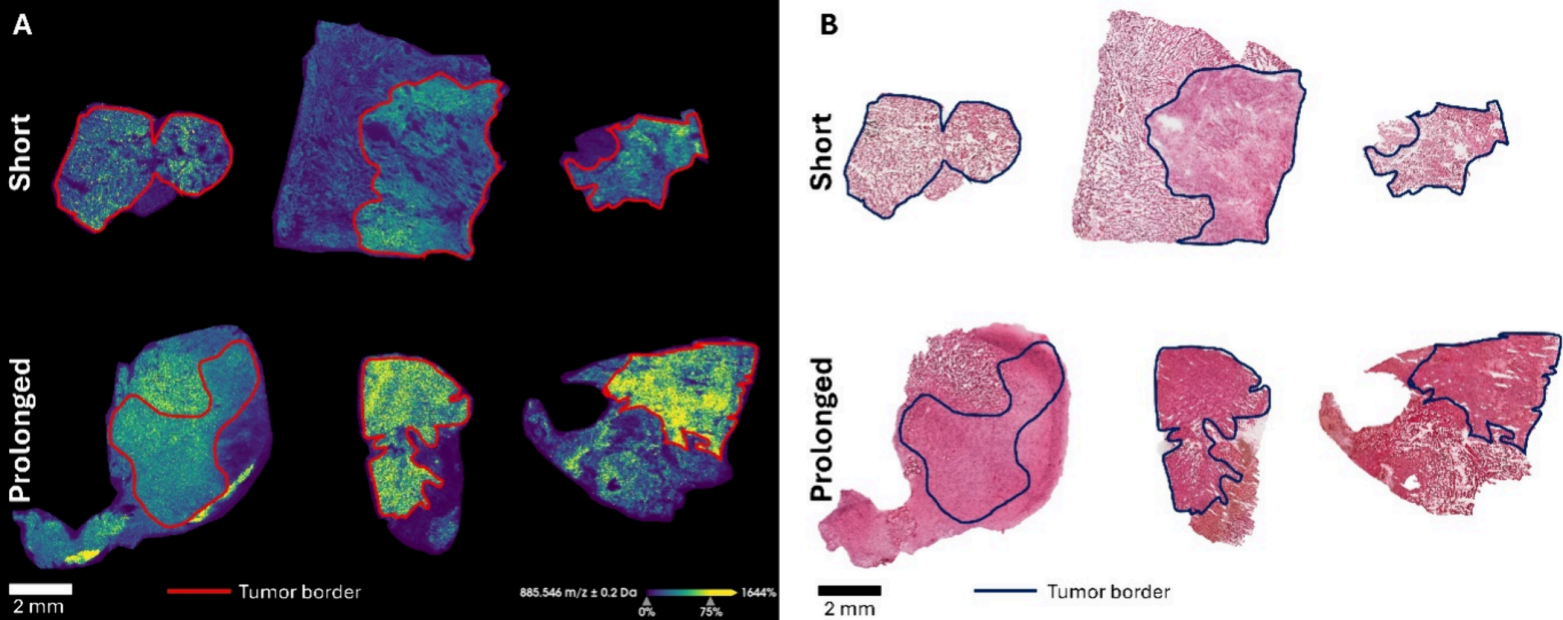
(A) MALDI-MSI of *m*/*z* 885.546 [PI (38:4)]-H in six GBM sections normalized by the norharmane matrix peak at *m*/*z* 333.147. *M*/*z* 885.546 resembles the tumor region and is indicated by red. (B) H&E-stained sections after MALDI-MSI measurement. The tumor border is indicated in black.

The REIMS workflow involves burning the samples blindly, without prior knowledge of the tissue’s specific histological features at each burning point. That means that the spectral profiles provided are based on heterogeneous areas that may include cancerous or inflammatory cells and necrotic or healthy tissues among others. To validate that the discriminating *m*/*z* values identified by REIMS are indeed associated with specific tumor regions, MALDI-MSI was employed. This approach allowed for the spatial correlation of *m*/*z* values with histologically defined areas within the tumor, confirming the lipidomic diversity and allowing precise characterization of GBM heterogeneity (Figure 2a). Here, *m*/*z* 885.54 and the H&E-stained sections (Figure 2b) were used to annotate the viable tumor region.^41^ In the negative ion mode, *m*/*z* 885.5 has been previously identified as PI (38:4)^−H^ based on the exact mass and existing literature, with Eberlin et al. reporting it in viable regions of GBM.^42^ Only the annotated tumor regions were used to find discriminating features between short-term and prolonged survival by using a receiver operating characteristic (ROC) analysis. An area under the curve (AUC) of >0.7 was used as discrimination quality measurement for the spectra produced by MALDI. Despite being a relatively modest threshold, this value was chosen to make sure that most positive cases are identified, even at the cost of increasing false positives, which could subsequently be examined and assessed for their significance. Moreover, the choice of a 0.7 threshold aligns with previous studies that employ ROC analysis in similar settings, where the goal is to identify an initial set of discriminators.^43−45^ In total, 42 and 33 discriminating features were found for short-term and prolonged survival, respectively (Supplementary Table S4). Comparing the discriminating features for REIMS and MALDI, several *m*/*z* values could be found in both modalities. The values derived from REIMS data were listed from observing the LDA loading plots, whereas for the ones found on MALDI data, their presence in the viable tumor regions and intensity was compared. Table 1 shows that in both REIMS and MALDI-MSI, the analytes PC (36:5)^−H^, PC (38:5)^−H^, PE (35:3)^−H^, PE (40:4)^−H^, and SM (42:2;2)^−CH^_3_ and one unidentified compound (*m*/*z* 752.520) were more prevalent in the short-term survival patients. For prolonged survival, we mainly detected PI (38:4)^−H^ and PI (38:3)^−H^ and four unidentified compounds (*m*/*z* 278.843, *m*/*z* 697.335, *m*/*z* 698.346, and *m*/*z* 737.405) that were responsible for the classification. The distribution of PC (38:5)^−H^ and the unidentified compound *m*/*z* 697.363, each more abundant in short-term and prolonged survivors, respectively, is visualized in Supplementary Figure S3.

**Table 1:** Discriminating *m*/*z* Features for Short-Term and Prolonged Survival Found in Both REIMS and MALDI-MSI^a^

**short survival**	**ID**	**adduct**	**prolonged survival**	**ID**	**adduct**
726.586	PE (35:3)	[M-H]^−^	278.843		
752.520			697.335		
778.505	PC (36:5)	[M-H]^−^	698.346		
794.516	PE (40:4)	[M-H]^−^	737.405		
797.651	SM (42:2)	[M-CH_3_]^−^	885.531	PI (38:4)	[M-H]^−^
806.538	PC (38:5)	[M-H]^−^	887.552	PI (38:3)	[M-H]^−^

a The *m*/*z* + 0.2 Da value is based on the MALDI-MSI measurement. LC-MS/MS is used for the identification of the analyte.

### Proteomic Profiling of Glioblastoma Tumor Regions

**Figure 3:**
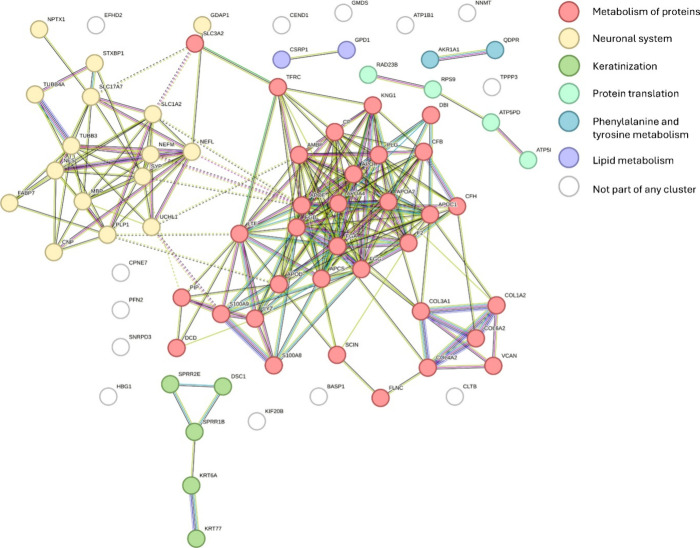
Pathway analysis of proteins significantly altered in both survival groups. Two main clusters reveal that the proteins are mainly involved in the metabolism of proteins (red) and the neuronal system (yellow).

Here, we performed proteomic profiling of glioblastoma tumor regions to gain insights into the proteomic differences between the short-term and prolonged survivors. Using LMD, the distinct tumor regions were precisely isolated, followed by proteomic analysis using LC-MS/MS. This approach allowed us to identify protein expression patterns unique to the survival groups. On a consecutive section to the MALDI-MSI measured section, 1 mm^2^ was extracted to analyze the differences between the tumor microenvironments. After proteomic extraction and acquisition, a total of 1387 proteins were identified. Proteins were significantly altered when a fold change of 1.5 took place (abundance ratio: log2 ≥ 0.58 for upregulation and ≤ −0.58 for downregulation) and an abundance ratio adjusted the *p*-value of ≤0.05. Also, the protein had to be present in at least two samples per group. A total of 79 significantly altered proteins were found and are shown in Supplementary Table S5. Sixty-six proteins showed upregulation in short-term survivors compared to prolonged survivors in comparison to the 13 upregulated proteins in prolonged survivors to short-term survivors. Performing a system biology analysis using Cytoscape and Reactome (Supplementary Figure S4 and Supplementary Table S6) shows that the significantly altered proteins are associated with several pathways,^46^ such as glial cell differentiation and mainly axon myelination (myelin basic protein (MBP), myelin proteolipid protein (PLP1), and 2′,3′-cyclic-nucleotide 3′-phosphodiesterase (CNP)). Fibrinogen alpha (FGA), beta (FGB), and gamma (FGG) chain protein, kininogen-1 (KNG1), plasminogen (PLG), collagen alpha-1 (III) (COL3A1), collagen alpha-2 (I) (COL1A2), and collagen alpha-2 (IV) (COL4A2) chain proteins are upregulated in short-term survival, and these are linked to β1, β2, and β3 integrin cell surface interactions. Interestingly, among the significantly altered proteins, an upregulation of apolipoprotein A-IV (APOA4), apolipoprotein E (APOE), serum amyloid P-component (APCS), lactotransferrin (LTF), and lysosome C (LYZ) in short-term survival is shown. Utilizing Reactome pathway analysis, these proteins show high relevance in amyloid β plaque formation. Proteins overexpressed in the prolonged survival group were linked to the NOTCH pathway (fatty acid-binding protein (FABP7), the astrocytic glutamate–glutamine uptake and metabolism (excitatory amino acid transporter 2 (SLC1A2)), and the glycerophospholipid biosynthesis (acyl-CoA-binding protein (ACBP), copine-7 (CPNE7), and nestin (NES)). Using StringDB, a full network overview of all significantly altered proteins can be found in Figure 3. Here, six different clusters can be seen, which resemble the metabolism of proteins (red), neuronal system (yellow), keratinization (lime green), protein translation (green), phenylalanine and tyrosine metabolism (blue), and lipid metabolism (purple).

## Discussion

In this study, we successfully developed a survival prediction model for IDH^WT^ GBM utilizing REIMS. Our findings reveal that the lipidomic profiles detected via REIMS and validated through MALDI-MSI exhibit significant spatial heterogeneity within the GBM section. Here, the feasibility of predicting survival times quasi-instantaneously by employing a PCA-LDA model based on the REIMS measurements is demonstrated. Furthermore, proteomic data analysis provided correlations with previously published research and discovered pathways relevant to patient survival, reinforcing the biological relevance of these findings. This study has enabled us to develop a robust predictive model, highlighting the potential for REIMS in enhancing the accuracy of overall survival predictions and thereby contributing to more personalized treatment strategies for GBM patients.

While our approach offers insights into intraoperative GBM research, already established methods used for survival prediction in GBM also need to be considered, such as MRI-based radiomics, genomic profiling, and traditional histopathological analysis.^47−49^ Studies by Kickingereder et al. in MRI-based radiomics and Zhang et al. in genomic profiling have already proven effective in providing valuable prognostic information for GBM patients.^50,51^ These methods focus on distinct biological and imaging features and can offer a comprehensive view of patient outcomes. REIMS could serve as an additional, complementary technique that provides real-time molecular insights during surgery, potentially filling an important gap in intraoperative decision-making.^27^ REIMS offers the advantage of rapid analysis without the need for extensive sample preparation. This ability to provide immediate feedback could be particularly valuable in guiding surgical decisions. There is a significant need for optimized intraoperative systems that provide instant tissue characterization without disrupting standard surgical procedures. REIMS has already proven effective in cancer research, including tumor margin detection for skin, breast, and cervical cancers.^52,53^ This study adds to its value, demonstrating that REIMS-generated models can differentiate patients with different survival outcomes based on their molecular profiles with 96.15% accuracy. Here, we also address a more challenging task: differentiating among GBM tissues with slight molecular variations that impact patient survival, rather than focusing solely on distinguishing between healthy and cancerous tissues.

The inclusion of 28 patients in the PCA/LDA REIMS model was determined to provide a balance between statistical reliability and practical feasibility, given the rarity of IDH^WT^ GBM biopsies and the complexity of intraoperative sampling. While larger cohorts are ideal for developing generalized models, studies often rely on smaller sample sizes due to the logistical challenges of collecting and analyzing high-quality data in a controlled research environment.^54,55^ Additionally, the use of LDA helps optimize the predictive power of smaller data sets by focusing on the most discriminative features of the data.^56^ Despite these considerations, we conducted an additional analysis to further validate the adequacy of our sample size. Research by Gan and Qi has demonstrated that while increasing the sample size can initially enhance model performance, a saturation point is often reached where additional data provide minimal benefits and may even reduce clarity due to overclustering or redundancy.^57^ To explore this, we utilized an alternative data set comprising glioblastoma tumor and normal post-mortem brain tissue samples, where survival outcomes were unavailable but similar classification models could be applied. Model accuracy was assessed across sample sizes ranging from 6 to 76 samples (Supplementary Table S7).^27^ Notably, in this alternative data set, the correct classification rate peaked at 24 samples, beyond which no significant improvements were observed, illustrating a plateau effect (Supplementary Figure S5). As this study is an initial proof of concept, the sample size is sufficient to demonstrate the feasibility of using REIMS for survival prediction while providing a foundation for larger multicenter validation studies in the future.

While past studies have primarily focused on distinguishing between healthy and cancerous tissues, where differences are more pronounced, our study addresses the more challenging task of differentiating among GBM tissues with slight molecular variations that impact patient survival.^58−60^ Moreover, previous works often relied solely on discriminators based on intensity or the mere presence or absence of certain compounds in tissue classes.^59,60^ In this case, even though the mass spectra showed visual differences upon inspection, differentiation was based on the ratios of compounds, a more complex analysis that underscores REIMS’s capability to detect subtle molecular differences. This approach highlights the potential of REIMS to identify key molecular variations that could impact prognosis and inform treatment strategies for GBM patients.

Despite these positive outcomes, the complex nature of brain tissues, especially highly heterogeneous GBM, may negatively influence discrimination accuracy.^61^ The fact that the REIMS workflow does not require sample preparation is an advantage; however, it results in a lack of control over the tissue. A GBM biopsy from a tumor resection is large enough to contain various types of tissues, and the burning point is usually set blindly.^61^ This heterogeneity can significantly impact the analysis and interpretation of biopsy results, as each tissue type has distinct molecular and histological characteristics. This raises the question of whether the molecular profiles are based on the cancerous part of the tumor, necrotic tissue, or infiltrated healthy regions.

In clinical settings, the heterogeneity of glioblastoma tissues can present a challenge for reliable classification. To address this, we propose a majority-vote approach, where multiple incisions from different tumor areas are analyzed, and classification is based on the majority outcome (e.g., 4 out of 5 samples aligning). Such an approach could account for local heterogeneity in clinical applications, aligning with standard diagnostic practices and making the model more adaptable for real-world intraoperative use.

To address the limitation of this study being conducted outside clinical settings, we employed MALDI-MSI to account for tumor microenvironment heterogeneity and to differentiate necrotic and viable regions as well as anatomical variations. A histopathological assessment can confirm the active tumor parts, and after their alignment with the MSI data, relevant mass peaks can be identified. This workflow integrates both modalities to cover each other’s weaknesses, ensuring the best possible results through comprehensive correlation of information. This hybrid approach ensures the most accurate and complete data, which is crucial for developing reliable and precise predictive models. Even after optimizing REIMS models, it was expected that the discriminators listed in the loading plots and those identified by MALDI would not perfectly match. Understanding that REIMS and MALDI-MSI are different ionization methods, some differences in the discriminants detected were anticipated.^62^ REIMS tends to cause more fragmentation due to its highly energetic ionization process, which should be considered when interpreting lipids.^63^ Although direct comparisons between REIMS and MALDI-MSI are still very limited, the existing studies indicate that both techniques are capable of ionizing and detecting similar lipid species.^64^ In REIMS, intact lipids, the few that remain in their complete, unfragmented state, will be observed at a lower intensity and will not completely align with the list of intact lipids identified by MALDI-MSI. Nevertheless, closely examining the fragments could still strengthen the lipidome identification. For example, REIMS data revealed numerous discriminators in the lower mass range, which have been previously identified in the literature as fatty acids and are likely products of phospholipid class fragmentation.^65,66^ Also, fragmentation may enhance the sensitivity of REIMS by revealing subtle molecular variations that would otherwise be overlooked, but it also requires thorough analysis to identify the biological relevance of the fragments and how they contribute to predictive modeling. Future work refining REIMS protocols to reduce fragmentation and correlate findings with MALDI-MSI and other techniques could clarify discrepancies and improve biomarker accuracy. As the model is based on existing data, expanding the database to include tissue heterogeneity and patient-specific characteristics, including genetic features, will improve its reliability.

Furthermore, the discriminators provided based on REIMS data are listed by their contribution to separating the classes, so it is easier to prioritize follow-up research. When accurately identified, these biomarkers can be used to predict patient outcomes or guide treatment strategies based on prognosis. O6-methylguanine DNA methyltransferase (MGMT) promoter methylation and IDH1 mutation were recently identified as molecular biomarkers for GBM, now regarded as favorable prognostic indicators for overall survival.^67,68^ IDH1 mutation is an exclusion criterion to call a malignant glioma a glioblastoma according to the WHO 2021 criteria. On the contrary, key signaling pathways such as PI3K/AKT/mTOR and Wnt tend to hyperactivate in GBM cases and worsen prognosis by aiding cancer cell proliferation.^69^ Likewise, discriminators identified through loading plots can be verified by MSI and serve as a starting point for biomarker research, concluding that REIMS-derived information with MSI techniques can improve image analysis precision. The confidence in identifying lipid-related pathways using REIMS and MALDI-MSI must be considered. While both techniques are highly effective for rapid lipid profiling and tissue screening, their ability to comprehensively identify biological pathways is limited compared to more detailed approaches such as LC-MS/MS-based proteomics.^70,71^ The pathway coverage in REIMS and MALDI is less extensive, primarily focusing on lipid species, yet these techniques can still provide valuable insights into the lipid composition and spatial distribution of molecules within samples.^72,73^ However, it is important to recognize that the broader pathway identification, which offers deeper proteomic and metabolomic profiling, may not be fully captured through REIMS and MALDI alone. Consequently, the pathway-level conclusions derived from these lipid-specific techniques should be interpreted with caution. This limitation underscores the rationale for incorporating proteomic data in our study, which provided more comprehensive biological insights and allowed for a more robust interpretation of the molecular pathways involved. The proteomic analysis further substantiated the lipidomic findings via REIMS and MALDI-MSI, revealing several key biomarkers and pathways associated with survival outcomes in GBM patients. Notably, we identified differential expressions of proteins involved in the metabolism of proteins, the neuronal system, and lipid metabolism. The data showed that MBP, PLP1, and CNP were notably upregulated in the short-term survival group. These proteins are intrinsically linked to the process of axon myelination, a crucial aspect of neuronal function and integrity.^74,75^ The elevated levels of these myelin-related proteins may be associated with upregulated lipid profiles, particularly involving phosphatidylcholine (PC) and phosphatidylethanolamine (PE), which play vital roles in membrane stability and signaling.^76,77^ In GBM, disrupted lipid metabolism can affect signaling pathways such as those mediated by phosphatidylinositol 3-kinase (PI3K), which is crucial for cell survival and growth, potentially leading to aggressive tumor behavior.^78^ Additionally, glioma cells have shown to migrate along myelin-rich white matter tracts, leveraging the lipid composition of myelin to facilitate their movement and invasion into surrounding brain tissues. For short-term survivors, this may indicate a disturbance in the myelination process due to the aggressive infiltration of GBM cells into white matter tracts, leading to a more rapid disease progression and a compromised neural environment.^79−81^ It has already been described that myelin damage could intensify the neurological decline, contributing to the reduced survival time observed in these patients.^82^ In addition to the upregulation of myelination-related proteins, our proteomic analysis revealed a significant upregulation of several extracellular matrix and coagulation-related proteins in short-term GBM survivors, including FGA, FGB, FGG, KNG1, PLG, COL3A1, COL1A2, and COL4A2. These proteins interact with key lipids such as sphingomyelin (SM) and phosphatidylinositol (PI), which are essential for maintaining membrane integrity and facilitating cell signaling, both of which are critical for tumor growth and patient survival.^83,84^ Research has shown that these proteins are associated with β1, β2, and β3 integrin cell surface interactions, which are critical for cell adhesion, migration, and signaling.^85^ The upregulation of these proteins suggests a heightened state of extracellular matrix remodeling and integrin-mediated signaling in short-term survivors, potentially contributing to the aggressive invasion and proliferation of GBM cells.^86^ Studies have also shown that β1 integrin downregulation shows a less aggressive phenotype in subgroups of patients with breast cancer.^87^ Interestingly, besides the upregulation of the β-integrins in the short-term survivors, research also shows that elevated β1 integrin levels promote glioma cell proliferation by negatively regulating the NOTCH pathway.^88^ The downregulation of the NOTCH pathway in short-term survivors can indicate why a significant difference between the short-term and prolonged survivors in the proteomic data was observed. Studies have shown that targeting both the β-integrins and the NOTCH pathway can disrupt these processes, potentially improving survival times for GBM patients.^89,90^ Moreover, alterations in glycerophospholipid metabolism, including changes in levels of PC, PE, and PI, also play a role in the pathophysiology of GBM, influencing tumor growth and patient survival as previously mentioned.^77,78,84^ Dysregulated lipid pathways, particularly those involving phospholipases that release fatty acids from glycerophospholipids, can activate prosurvival signaling cascades and contribute to the aggressive phenotype of GBM.^91^ Alterations in ACBP, crucial for lipid metabolism and transport, result in dysregulated lipid homeostasis and in turn sustain GBM invasion.^92,93^ Previous research has already proven that SLC1A2 enhances the glutamate release from glioma cells, which contributes to tumor-associated necrosis. These interactions between lipids and proteins not only provide critical insights into the molecular mechanisms driving the observed lipidomic heterogeneity but also underscore their impact on survival, thereby reinforcing the potential clinical utility of our predictive model. Lipid–protein interactions could potentially serve as therapeutic targets, offering new avenues for improving treatment strategies in GBM.

In conclusion, this research shows the potential use of electrocautery and REIMS as a quasi-instantaneous classification tool for predicting the survival time of IDH^WT^ GBM patients. Similar lipidomic profiles found between REIMS and MALDI-MSI in the tumor regions were revealed by MALDI-MSI. Using LMD, we were able to perform proteomics on the tumor regions and dive deeper into the survival outcome differences by looking at significantly altered proteins. Most altered proteins have earlier been proven to affect cancer aggressiveness and survival times in GBM and other cancers and were mainly used for confirmation. However, the upregulation of β2 integrin also indicates a decrease in overall survival and could be a potential biomarker that can therapeutically be targeted. Despite the limitations in sensitivity, this study already demonstrates the feasibility of integrating REIMS into intraoperative diagnostics, offering real-time insights into glioblastoma patient survival that can ultimately guide personalized treatment strategies. Future advancements in database expansion and technique refinement hold promise for improving the reliability and clinical utility of this predictive approach. For future clinical translation, we propose a multiphase roadmap to eventually integrate REIMS into routine glioblastoma surgeries. First, further validation studies in larger, multicenter patient cohorts will establish the reliability of REIMS predictions across diverse populations. Following this, regulatory approvals and standardization of REIMS instrumentation and protocols will ensure the method’s reproducibility in clinical settings.^94^ The next phase would involve combining REIMS data with other established prognostic methods, such as MRI-based radiomics and genomic profiling, to develop a comprehensive diagnostic platform.^95^ Finally, training surgeons and oncologists in the effective use of REIMS for intraoperative decision-making will be crucial to seamlessly incorporate this technique into standard care.

## Materials and Methods

### Patient Samples and Characteristics

Ethical approval was gained from the UZ/KU Leuven Ethical Review Committee of University Hospitals Leuven (Gasthuisberg, Leuven, Belgium) with reference number S60290, and the project was registered under the UZ Leuven Tissue Bank. From the cohort, 91 GBM samples were collected. The status of the IDH gene in 13 patients was either IDH^M^ or unknown; these patients were excluded from the study. The remaining 78 patients gave consent for the resected tissue samples to be included in the research. Confirmed overall survival information was provided for 45 out of the 78 patients, which was used for the REIMS model categorization and their manual validation (Supplementary Table S1). The study included patients who had undergone surgery in UZ Leuven between September 2018 and September 2023. Samples were obtained from enrolled patients undergoing surgery under total intravenous general anesthesia and 5-ALA guidance. Most patients with newly diagnosed GBM were given a single oral dose of 5-ALA (Gliolan 20 mg/kg, NX Development Corp., Lexington, KY) 3 h prior to induction of anesthesia. The dose was dependent on each patient’s BMI and overall medical status. All collected samples were stored at −80 °C with no additional processing before further analysis.

### REIMS Analysis

All parts of the REIMS procedure took place inside a laminar-flow biosafety cabinet (Biowizard Xtra Line, Kojair Blue Series Technologies) where a silicone return electrode mat was placed. The surface of the mat was covered with low-lint and high-absorbency inert wipes (Kimtech, Kimberly Clark) and soaked in deionized water to provide an uninterrupted connection between the mat and the sample. Before REIMS analysis, human GBM samples were placed on the electrically conductive mat and thawed to room temperature. For electrocauterization, an electrical surgical knife was used, previously described as the iKnife.^72^ This surgical knife was connected to a Xevo G2-XS quadrupole time-of-flight mass spectrometer equipped with a REIMS interface (Waters Corporation). Depending on the tissue size, consistency, and signal quality, 1–5 incisions were made per GBM sample using a monopole electrosurgical knife for approximately 1–2 s in cut mode operated by a FORCE FX electrical heat generator (Covidien Ltd.) at a power of 15 W. The generated vapors were transferred into the REIMS interface through a polymer tubing (Tygon) of 2 m that was regularly replaced. For internal lock-mass calibration, leucine-enkephalin (Sigma-Aldrich) was dissolved in 2-propanol (BioSolve) and continuously infused at 150 μL/min through an external syringe. As for the mass spectrometer settings, the cone voltage was set to 40 V, the heater bias to 80 V, and the StepWave RF amplitude to 300 V, and the collision cell RF was operated at 400 V offset. The mass spectrometer was operated in negative ionization and sensitivity mode. Data acquisition was performed at a mass range of *m*/*z* 100–1000 with a scan time of 1 s.

### REIMS Data Analysis

Data acquisition for REIMS spectra was supported by MassLynx 4.1 MS software (Waters Corporation) through which background noise was subtracted and peaks got centralized. Abstract Model Builder (AMX) (AMX, version 0.9.2092.0, Waters Research Center, Hungary) was then used to process all raw mass spectrometric data collected on REIMS. AMX was also employed for the recognition model construction and classification. The mean mass spectra of each incision were summed based on their intensities to produce a single profile since spectrum interpretation was set at one unique spectrum per sampling point. All spectra were binned with an advanced bin of 0.2 and a mass range of *m*/*z* 250–1000. Lock-mass correction and background subtraction were applied to improve the mass accuracy and precision of measured ions before building the models. Activating them ensures that unwanted signals, typically originating from noise or interfering compounds, are removed from the MSI data. Subsequently, systematic variations in the signal intensity across the data set were corrected, through normalization, to facilitate meaningful comparison and interpretation of different spatial regions. Spectra were normalized by dividing each of their points by the average intensity to get them all in the same order. Multivariate analysis was established using PCA/LDA. PCA was performed with a maximum of *n* = 30 components and LDA with *n* – 1 components where *n* corresponds to the number of variables introduced. Cross-validation tests were performed by using 20%-out cross-validation, which is the proposed method to validate models created based on multivariate statistics. This type of cross-validation randomly selects 20% of the spectra to exclude from the training set and builds the model on the remaining 80%. The process is then repeated 5 times to include all obtained spectra. Outliers, meaning cases that were not an adequate fit to either of the groups, were not included as an option in the validation process, forcing all burning points to one of the two categories. The PCA/LDA model created using AMX Abstract Model Builder was exported into AMX Recognition v0.9.2092.0 and used in postprocessing mode to directly categorize samples not included in the model, manually testing its efficacy.

### MALDI-MSI Analysis

Prior to MALDI-MSI analysis, human GBM was sectioned at 10 μm thickness using a cryotome (Leica 1850 UV, Leica) and thaw-mounted on indium tin oxide (ITO) microscope slides (Delta Technologies). Norharmane (Sigma-Aldrich) was dissolved at 7 mg/mL in 2:1 chloroform and methanol (BioSolve) and homogeneously sprayed using an HTX TM-sprayer (HTX Technologies). The sprayer settings are provided in the Supporting Information. MALDI-MSI was performed in negative ionization mode on a RapifleX MALDI Tissuetyper TOF mass spectrometer (Bruker Daltonik). The acquisition was performed with an accumulation of 100 shots per pixel at a 5 kHz laser frequency using a Nd:YAG 355 nm SmartBeam 3D laser (Ekspla). Mass spectra were collected at a mass range of *m*/*z* 100–1000 at a spatial resolution of 30 × 30 μm. Before the imaging experiments, time-of-flight calibration was performed using red phosphorus. Afterward, hematoxylin and eosin (H&E) staining was performed, and the protocol used can be found in the Supporting Information.

### MALDI-MSI Data Analysis

FlexImaging v5.0 (Bruker Daltonik, Bremen, Germany) was used for the initial process of the mass spectrometry imaging data acquired. Prior to data analysis, FlexAnalysis (Bruker Daltonik, Bremen, Germany) was used to recalibrate the MSI data to correct for any inherent *m*/*z* deviations. Prominent peaks within the acquired mass spectra corresponding to norharmane were used to recalibrate. The *m*/*z* values used as reference points were norharmane matrix-related peaks at *m*/*z* 167.061, 203.038, and 333.114 and an endogenous brain lipid at *m*/*z* 885.5499. After recalibration, the MALDI-MSI data were analyzed using SCiLS Lab MVS 2024a software (SCiLS GmbH). Peak picking was performed in mMass v5.5.0 by using a signal-to-noise threshold of 3.0 and a picking height of 80 and by applying a baseline, smoothing, and deisotoping.^96^ The data were normalized using the peak area of a norharmane peak at *m*/*z* 333.114. Unsupervised multivariate analysis was carried out (PCA), and receiver operating characteristic (ROC) area under the curve (AUC) analysis was performed to find ions that discriminated between the annotated viable tumor regions. A threshold cutoff value of >0.7 was used. When all MSI data were imported into the statistical analysis software, the viable parts were annotated based on the H&E images and the said mass peaks, ensuring that the statistical analysis was performed based only on the tumor areas.

### Lipid Identification via LC-MS/MS

Tumor regions from human GBM sections were dissected via laser-capture microdissection (LMD) and captured in 100 μL of methanol (BioSolve). Lipids were extracted by adding 400 μL of methyl-*tert*-butyl-ether (MTBE) (Sigma-Aldrich) to the dissected material, vortexed for 1 min, and placed in a thermoshaker for 1 h at 20 °C at 950 rpm. Following this, 100 μL of water (BioSolve) was added, vortexed for 1 min, and centrifuged at 1000*g* for 10 min in an Eppendorf centrifuge 5353 (Eppendorf). The lipid containing the upper organic fraction was transferred to a new tube. A re-extraction of lipids was performed to the bottom fraction by adding 300 μL of MTBE, 40 μL of methanol, and 30 μL of water. The sample was again vortexed for 1 min and centrifuged for 10 min at 1000*g*, and the upper organic layer was combined with the previously extracted organic layer. Afterward, the organic fraction was dried in a vacuum centrifuge (Heto Lab), and the dried fraction was reconstituted in 50 μL of 1:1 2-propanol:acetonitrile (BioSolve) and used for LC-MS/MS (Vanquish UHPLC, HypersilGOLD 100 × 2.1 mm, Orbitrap Exploris480, Thermo Fisher). The lipidomic analysis was based on a generic lipidomics protocol in negative ionization mode.^97^ The lipid identification can be found in the Supporting Information.^98^

### Proteomics

Consecutive sections to the six MALDI-MSI sections (three short survival and three prolonged survival) were subjected to LMD. One mm^2^ of the tumor region was dissected and captured in 20 μL of 50 mM ammonium bicarbonate (Sigma-Aldrich, The Netherlands). A volume of 2.2 μL of 0.1% Rapigest was added to the sample solution to aid in protein solubility, followed by incubation at 21 °C for 10 min at 800 rpm. To reduce disulfide bonds, 1.3 μL of 10 mM DTT was added and the solution was incubated at 56 °C for 40 min at 800 rpm. To prevent disulfide bond reformation, cysteines were alkylated with 1.4 μL of 20 mM IAM, and the mixture was incubated at room temperature for 30 min. Subsequently, 1.4 μL of 10 mM DTT was added and incubated at room temperature for 10 min at 800 rpm. Then, 1 μL of 0.5 μg/μL trypsin was added for overnight digestion at 37 °C and 800 rpm. Following this, 0.3 μL of trypsin and 115 μL of ACN were added for a final digestion at 37 °C for 3 h at 800 rpm. To terminate the reaction, 6 μL of 10% TFA was added and the mixture was incubated for 45 min at 37 °C and 800 rpm. The samples were centrifuged at 15,000*g* for 15 min at 4 °C. The supernatant was then transferred to a new vial and concentrated by vacuum centrifuging to a final volume of 30 μL.

Peptide separation was performed using a Thermo Scientific (Dionex) Ultimate 3000 Rapid Separation UHPLC system equipped with a Thermo Scientific Acclaim PepMap C18 analytical column (150 × 0.75 mm, 3 μm). Peptide samples were desalted on an online C18 trapping column. Following desalting, peptides were chromatographically separated on the analytical column using a 110 min gradient from 4 to 32% ACN with 0.1% FA at a flow rate of 300 nL/min. The UHPLC system was connected to a Q Exactive HF mass spectrometer (Thermo Scientific). Data-dependent acquisition (DDA) settings were as follows: MS1 scans were conducted between 250 and 1250 *m*/*z* at a resolution of 120,000, followed by MS2 scans of the top 15 most intense ions at a resolution of 15,000.

### Protein Identification and Analyses

RAW data obtained via LC-MS/MS analysis were analyzed using Proteome Discoverer V3.0 (Thermo Scientific) LFQ quantification. Using the *Homo sapiens* protein database (UniProt taxonomy ID 9606), the following parameters were applied for the protein database search: trypsin was selected as the enzyme, allowing up to two missed cleavage sites, with a minimum peptide length of six amino acids. The precursor mass range was set between 350 and 5000 Da. The mass tolerance was specified as 10 ppm for precursors and 0.02 Da for fragments. Dynamic modifications included acetylation of the N-terminus and methionine oxidation, while carbamidomethylation was used as a static modification. A strict false discovery rate (FDR) of 0.01 was enforced to ensure high confidence in identification. Protein accession numbers were utilized to determine protein-encoding gene names via the UniProtKB database. A protein interaction network was created using the STRING database (version 12.0).^99^ The potential interaction pathways were generated by using Reactome (version 88).^100^

## Supporting Information Available

Matrix deposition; H&E staining; lipid identification; ST1: patient characteristics; ST2: loading discriminants in REIMS; ST3: REIMS classification of unknown samples; ST4: discriminating values in MALDI-MSI; ST5: significantly altered proteins in short-term and prolonged survival; ST6: pathway analysis via Reactome; ST7: models’ parameters; SF1: PCA components and correct classification rate; SF2: PCA GBM overall survival; SF3: MALDI-MSI of short-term and prolonged survival GBM sections; SF4: Reactome of significantly altered pathways; SF5: sample size vs classification accuracy (PDF)
