# Supplementary material for: Integrating Rapid Evaporative Ionization Mass Spectrometry Classification with Matrix-Assisted Laser Desorption Ionization Mass Spectrometry Imaging and Liquid Chromatography-Tandem Mass Spectrometry to Unveil Glioblastoma Overall Survival Prediction
Source: ACS Chem Neurosci. 2025 Feb 26;16(6):1021–33. doi: 10.1021/acschemneuro.4c00463 (PMC11926789; doi:10.1021/acschemneuro.4c00463)
Supplement: Supplementary file 1 — cn4c00463_si_001.pdf [file cn4c00463_si_001.pdf]

Supplemental Information:

Integrating REIMS Classification with MALDI-MSI and LC-MS/MS to Unveil Glioblastoma Overall Survival Prediction

Tim F.E. Hendriks[1#], Angeliki Birmpili[1#], Steven de Vleeschouwer[2], Ron M.A. Heeren[1], Eva Cuypers[1*]

[1]The Maastricht MultiModal Molecular Imaging (M4I) Institute, Division of Imaging Mass Spectrometry (IMS), Maastricht University, 6229 ER Maastricht, The Netherlands

[2]Department of Neurosurgery, UZ Leuven, and Laboratory for Experimental Neurosurgery and Neuroanatomy, Department of Neurosciences and Leuven Brain Institute (LBI), KU Leuven, 3000 Leuven, Belgium

[#]Contributed equally

*Corresponding author: e.cuypers@maastrichtuniversity.nl

Supplementary information content

Matrix deposition 3

Hematoxylin and eosin staining 3

Lipid identification 3

Supplementary table S1: Patient characteristics from the selected cohort. All patients have IDH^{WT}. 4

Supplementary table S2: Loading discriminant 1, top 50 *m/z* contributors defined via REIMS. 5

Supplementary table S3: Classifying REIMS burns of unknown samples in GBM survival model. 6

Supplementary table S4: Discriminating features of short-term and prolonged survival defined via MALDI-MSI. 7

Supplementary table S5: Significantly altered proteins between short and prolonged survival glioblastoma patients via LC-MS/MS proteomics. 8

Supplementary table S6: Pathway analysis via Reactome. 9

Supplementary table S7: Models' parameters. 10

Supplementary figure S1: Line chart illustrating the relationship between the number of PCA components and the correct classification rates of potential models. 11

Supplementary figure S2: PCA model classifying human GBM IV patient overall survival times. 11

Supplementary figure S3: MALDI-MSI (-) of short- and prolonged survivor glioblastoma sections. 12

Supplementary figure S4: Reactome overview of significantly altered pathways..13

Supplementary figure S5: Line chart illustrating the relationship between the sample size and classification accuracy for different-sized models. 14

Matrix deposition

Samples were sprayed with 7 mg/mL norharmane diluted in 2:1 CHCl3:MeOH using a 40mm spray nozzle. Spraying parameters for norharmane were as follows: temperature = 40°C, flow rate = 0.12 mL/min, velocity = 1200 mm/min, track spacing = 3 mm, gas flow = 2 L/min, N_2 gas pressure = 10 psi, nozzle height = 40 mm, drying time = 30 s, number of passes = 15, in a C-C pattern.

Hematoxylin and eosin staining

The H&E staining procedure was conducted as follows: the sections were hydrated in water for 1 minute, stained with hematoxylin for 3 minutes, and rinsed under running tap water for 3 minutes. Next, the slides were stained with eosin for 30 seconds and rinsed again under running tap water for 3 minutes. The slides were then immersed in 100% ethanol for 1 minute, washed with xylene for 2 minutes, and carefully mounted with Entellan and a coverslip, before being left to air-dry at room temperature. All optical images were captured in high resolution using the Leica AperioCS2 scanner (20x objective) with Aperio ImageScope software (version 12.4.3.5008) from Leica Biosystems Imaging (Germany).

Lipid identification

The lipid identification was performed with an Orbitrap Exploris480 mass spectrometer (Thermo Fisher Scientific, USA) running in data-dependent acquisition (DDA) negative ionization mode. Here, MS^1 data of *m/z* 200–1450 were acquired with a mass resolution of 60000 and an injection time of 65ms. In parallel, MS^2 data were acquired in the ion trap via collision-induced dissociation (CID) using an isolation window of 1.7 Da and a mass resolution of 30000. The lipid species in LC-MS were assigned using MS^1 and MS^2 spectra acquired from the DDA measurements in Lipostar2 version v2.1.2. Lipid identifications for MALDI-MSI were assigned by combining MS^1 *m/*z values measured via MALDI-MSI to the MS^1+MS^2 *m/z* values found in the LC-MS/MS measurements. The combination of MS^1 (MALDI) and MS^2 (LC-MS/MS) was used for identification in LipostarMSI v2.0.1, both using the LIPID MAPS database (3- and 4-star rating, Molecular Horizon, Bettona, PG, Italy). For the current study, we utilized the lipid identifications from our prior publication to ensure consistency and reliability of the lipid species identified. The detailed methodology of the lipid extraction, identification, and the criteria for lipid species confirmation are comprehensively described in the earlier publication [34].

Supplementary table S1: Patient characteristics from the selected cohort. All patients have IDH^{WT}.

	**Total population (*n*=45)**
**Sex**	
Female	14 (31%)
Male	31 (69%)
**Age at diagnosis**	
Median (IQR)	60 (51 – 69)
**Main location of the tumor**	
Frontal	12 (27%)
Occipital	1 (2%)
Parietal	13 (28%)
Temporal	19 (43%)
**Initial treatment**	
RT/TMZ followed by < 6 cycles of TMZ	22 (49%)
RT/TMZ followed by 6 cycles of TMZ	16 (36%)
RT/TMZ followed by > 6 cycles of TMZ	2 (4%)
Other treatment*	2 (4%)
No treatment	3 (7%)
**Overall survival time deceased**	
0 – 12 months	20 (44%)
13 – 24 months	16 (36%)
> 24 months	9 (20%)
**Genetic mutations**	
1p/19q codeletion	1
p53	19
GFAP	29
Ki-67	6
ATRX	23

* Other treatments administered to patients were Lomustine (1) and Dabrafenib (1).

Supplementary table S2: Loading discriminant 1, top 50 *m/z* contributors defined via REIMS.

**#**	***m/z* LD1 (+) Short survival**	**LD1 score (+) Short survival**	***m/z* LD1 (-) Prolonged survival**	**LD1 score (-) Prolonged survival**
**1**	255.3	0.207	283.3	-0.349
**2**	657.5	0.198	742.5	-0.223
**3**	305.3	0.154	278.9	-0.209
**4**	682.5	0.149	706.5	-0.160
**5**	276.1	0.129	687.5	-0.149
**6**	281.3	0.127	735.5	-0.144
**7**	747.5	0.108	697.5	-0.119
**8**	794.5	0.103	717.5	-0.112
**9**	766.5	0.102	743.5	-0.105
**10**	749.5	0.102	862.7	-0.104
**11**	750.5	0.092	576.3	-0.103
**12**	727.5	0.088	642.5	-0.101
**13**	253.3	0.082	283.1	-0.088
**14**	774.5	0.081	715.5	-0.083
**15**	331.3	0.080	718.5	-0.080
**16**	658.5	0.078	600.5	-0.079
**17**	684.5	0.075	693.5	-0.072
**18**	659.5	0.068	303.3	-0.072
**19**	671.5	0.067	688.5	-0.070
**20**	716.5	0.067	955.7	-0.069
**21**	617.3	0.065	708.5	-0.066
**22**	844.7	0.065	284.3	-0.064
**23**	776.5	0.064	707.5	-0.063
**24**	778.5	0.062	269.1	-0.061
**25**	265.1	0.060	580.3	-0.058
**26**	269.3	0.060	736.5	-0.058
**27**	773.5	0.058	602.5	-0.058
**28**	792.5	0.058	863.7	-0.054
**29**	790.5	0.058	460.3	-0.053
**30**	748.5	0.057	254.1	-0.053
**31**	375.3	0.056	365.3	-0.053
**32**	419.3	0.054	744.5	-0.052
**33**	683.5	0.053	258.1	-0.052
**34**	645.5	0.052	864.7	-0.051
**35**	767.5	0.052	268.1	-0.049
**36**	639.3	0.049	860.7	-0.049
**37**	772.5	0.048	885.5	-0.048
**38**	668.5	0.047	367.3	-0.048
**39**	751.5	0.047	874.7	-0.044
**40**	276.7	0.047	956.7	-0.044
**41**	652.3	0.046	737.5	-0.044
**42**	775.5	0.046	463.3	-0.044
**43**	686.5	0.046	255.1	-0.043
**44**	752.2	0.045	280.3	-0.042
**45**	797.5	0.044	887.7	-0.042
**46**	701.5	0.043	257.1	-0.041
**47**	417.3	0.043	252.1	-0.041
**48**	795.5	0.042	872.7	-0.040
**49**	806.5	0.042	953.7	-0.040
**50**	311.1	0.042	281.1	-0.040

Supplementary table S3: Classifying REIMS burns of unknown samples in GBM survival model.

Patient	Overall survival (months)	# burns	Classified as short (0-12 months)	Classified as prolonged (> 12 months)	Classified based on the majority of classified burns
**P1**	6.5	3	2	1	Short-term
**P2**	8.13	1	1	-	Short-term
**P3**	8.8	1	-	1	Long-term
**P4**	9.5	3	1	2	Long-term
**P5**	11.03	3	2	1	Short-term
**P6**	11.07	4	4	-	Short-term
**Total short**		15	**10 (66.7%)**	5 (33.3%)	**4 (66.7%)**
**P8**	12.5	3	3	-	Short-term
**P9**	12.5	3	3	-	Short-term
**P10**	12.6	3	-	3	Long-term
**P11**	13	5	-	5	Long-term
**P12**	13.8	3	2	1	Short-term
**P13**	15.6	1	-	1	Long-term
**P14**	19.1	3	3	-	Short-term
**P15**	22.7	9	1	8	Long-term
**P16**	24.0	11	3	8	Long-term
**P17**	27.7	3	-	3	Long-term
**P18**	50.2	5	-	5	Long-term
**Total long**		49	15 (30.6%)	**34 (69.4%)**	**7 (63.6%)**

Supplementary table S4: Discriminating features of short-term and prolonged survival defined via MALDI-MSI.

**#**	**m/z ± 0.2 Da ROC Short survival**	**AUC Short survival**	**m/z ± 0.2 Da ROC Long survival**	**AUC Long survival**
**1**	195.07	0.723	123.998	0.258
**2**	247.03	0.884	132.996	0.208
**3**	316.97	0.786	135.019	0.177
**4**	334.12	0.710	135.972	0.290
**5**	357.11	0.710	146.966	0.266
**6**	358.11	0.847	152.012	0.274
**7**	439.01	0.718	152.761	0.247
**8**	478.28	0.723	186.048	0.058
**9**	648.37	0.700	187.031	0.118
**10**	**726.53**	**0.767**	187.323	0.214
**11**	**752.09**	**0.704**	188.045	0.210
**12**	**778.51**	**0.807**	188.933	0.271
**13**	**794.52**	**0.746**	213.946	0.254
**14**	**797.62**	**0.756**	**278.848**	0.274
**15**	804.53	0.860	330.046	0.247
**16**	**806.54**	**0.950**	528.25	0.255
**17**	808.52	0.863	552.244	0.213
**18**	822.53	0.744	553.195	0.152
**19**	862.58	0.705	599.286	0.290
**20**	876.61	0.763	644.458	0.274
**21**	878.60	0.799	670.445	0.270
**22**	880.59	0.777	**697.329**	0.177
**23**	888.62	0.776	**698.353**	0.275
**24**	889.96	0.824	713.342	0.131
**25**	890.63	0.789	722.478	0.222
**26**	891.02	0.772	723.476	0.257
**27**	892.63	0.814	**737.393**	0.286
**28**	894.62	0.788	746.492	0.289
**29**	902.64	0.774	747.485	0.245
**30**	904.63	0.847	753.419	0.267
**31**	906.64	0.834	857.504	0.280
**32**	908.64	0.811	**885.546**	0.230
**33**	914.62	0.734	**887.982**	0.298
**34**	916.65	0.866		
**35**	918.65	0.915		
**36**	920.65	0.959		
**37**	922.65	0.875		
**38**	930.63	0.833		
**39**	931.60	0.737		
**40**	932.65	0.926		
**41**	934.66	0.946		
**42**	936.65	0.821		

Values in **bold** are both found as REIMS loading discriminant as well as MALDI-MSI discriminating factor for their corresponding survival time.

Supplementary table S5: Significantly altered proteins between short and prolonged survival glioblastoma patients via LC-MS/MS proteomics.

Upregulated short-term survival		Upregulated short-term survival		Upregulated prolonged survival	
**Protein**	**Abbreviation**	**Protein**	**Abbreviation**	**Protein**	**Abbreviation**
2',3'-cyclic-nucleotide 3'-phosphodiesterase	CNP	Immunoglobulin heavy constant gamma 3	IGHG3	ATP synthase subunit d, mitochondrial	ATP5PD
40S ribosomal protein S9	RPS9	Immunoglobulin heavy constant gamma 4	IGHG4	Acyl-CoA-binding protein	ACBP
4F2 cell-surface antigen heavy chain	SLC3A2	Immunoglobulin kappa variable 3-20	IGKV3-20	ATPase inhibitor, mitochondrial	ATP5IF1
Aldo-keto reductase family 1 member A1	AKR1A1	Keratin, type II cytoskeletal 1b	KRT77	Clathrin light chain B	CLTB
Apolipoprotein A-II	APOA2	Keratin, type II cytoskeletal 6A	KRT6A	Collagen alpha-2(VI) chain	COL6A2
Apolipoprotein A-IV	APOA4	Kinesin-like protein KIF20B	KIF20B	Copine-7	CPNE7
Apolipoprotein C-I	APOC1	Kininogen-1	KNG1	Excitatory amino acid transporter 2	SLC1A2
Apolipoprotein D	APOD	Lactotransferrin	LTF	Fatty acid-binding protein, brain	FABP7
Apolipoprotein E	APOE	Lysozyme C	LYZ	Nestin	NES
Beta-2-glycoprotein 1	APOH	Myelin basic protein	MBP	Nicotinamide N-methyltransferase	NNMT
Brain acid soluble protein 1	BASP1	Myelin proteolipid protein	PLP1	Small nuclear ribonucleoprotein Sm D3	SNRPD3
Cell cycle exit and neuronal differentiation protein 1	CEND1	Neurofilament light polypeptide	NEFL	Transferrin receptor protein 1	TFRC
Ceruloplasmin	CP	Neurofilament medium polypeptide	NEFM	UV excision repair protein RAD23 homolog B	RAD23B
Collagen alpha-1(III) chain	COL3A1	Neuronal pentraxin-1	NPTX1		
Collagen alpha-2(I) chain	COL1A2	Plasminogen	PLG		
Collagen alpha-2(IV) chain	COL4A2	Profilin-2	PFN2		
Complement factor B	CFB	Prolactin-inducible protein	PIP		
Complement factor H	CFH	Protein AMBP	AMBP		
Cornifin-B	SPRR1B	Protein S100-A8	S100A8		
Cysteine and glycine-rich protein 1	CSRP1	Protein S100-A9	S100A9		
Dermcidin	DCD	Prothrombin	F2		
Desmocollin-1	DSC1	Scinderin	SCIN		
Dihydropteridine reductase	QDPR	Serum amyloid P-component	APCS		
EF-hand domain-containing protein D2	EFHD2	Small proline-rich protein 2E	SPRR2E		
Fibrinogen alpha chain	FGA	Sodium/potassium-transporting ATPase subunit beta-1	ATP1B1		
Fibrinogen beta chain	FGB	Synaptophysin	SYP		
Fibrinogen gamma chain	FGG	Syntaxin-binding protein 1	STXBP1		
Filamin-C	FLNC	Tubulin beta-3 chain	TUBB3		
Ganglioside-induced differentiation-associated protein 1	GDAP1	Tubulin beta-4A chain	TUBB4A		
GDP-mannose 4,6 dehydratase	GMDS	Tubulin polymerization-promoting protein family member 3	TPPP3		
Glycerol-3-phosphate dehydrogenase [NAD(+)], cytoplasmic	GPD1	Ubiquitin carboxyl-terminal hydrolase isozyme L1	UCHL1		
Hemoglobin subunit gamma-1	HBG1	Versican core protein	VCAN		
Immunoglobulin heavy constant gamma 2	IGHG2	Vesicular glutamate transporter 1	SLC17A7		

Supplementary table S6: Pathway analysis via Reactome.

Pathway name	#Entities found	#Entities total	Entities ratio	Entities pValue	Entities FDR
Regulation of Complement cascade	10	139	0.008893	2.35E-08	1.09E-05
Complement cascade	10	156	0.009981	6.81E-08	1.58E-05
Regulation of Insulin-like Growth Factor (IGF) transport and uptake by Insulin-like Growth Factor Binding Proteins (IGFBPs)	9	127	0.008125	1.38E-07	2.13E-05
MyD88 deficiency (TLR2/4)	5	26	0.001663	8.00E-07	8.84E-05
IRAK4 deficiency (TLR2/4)	5	27	0.001727	9.61E-07	8.84E-05
Integrin cell surface interactions	7	86	0.005502	1.46E-06	1.12E-04
NR1H3 & NR1H2 regulate gene expression linked to cholesterol transport and efflux	6	66	0.004223	4.54E-06	3.00E-04
Post-translational protein phosphorylation	7	109	0.006974	6.82E-06	3.69E-04
Regulation of TLR by endogenous ligand	5	41	0.002623	7.24E-06	3.69E-04
Formation of Fibrin Clot (Clotting Cascade)	5	43	0.002751	9.09E-06	4.18E-04
Diseases associated with the TLR signaling cascade	5	47	0.003007	1.39E-05	5.28E-04
Diseases of Immune System	5	47	0.003007	1.39E-05	5.28E-04
NR1H2 and NR1H3-mediated signaling	6	85	0.005438	1.88E-05	6.58E-04
Common Pathway of Fibrin Clot Formation	4	25	0.001599	2.18E-05	7.13E-04
Amyloid fiber formation	6	89	0.005694	2.43E-05	7.13E-04
Platelet Aggregation (Plug Formation)	5	53	0.003391	2.46E-05	7.13E-04
Formation of the cornified envelope	7	138	0.008829	3.06E-05	8.27E-04
Plasma lipoprotein assembly	4	30	0.001919	4.42E-05	0.001098
Hemostasis	16	804	0.05144	4.58E-05	0.001098
Metal sequestration by antimicrobial proteins	3	13	8.32E-04	8.50E-05	0.001955
Chylomicron assembly	3	14	8.96E-04	1.06E-04	0.002325
ECM proteoglycans	5	79	0.005054	1.59E-04	0.00334
Chylomicron remodeling	3	17	0.001088	1.87E-04	0.003629
Collagen chain trimerization	4	44	0.002815	1.91E-04	0.003629
Extracellular matrix organization	9	328	0.020985	2.50E-04	0.004494
Innate Immune System	20	1347	0.08618	2.70E-04	0.004497
Scavenging by Class A Receptors	4	49	0.003135	2.87E-04	0.004497
Platelet degranulation	6	141	0.009021	2.95E-04	0.004497
GRB2:SOS provides linkage to MAPK signaling for Integrins	3	20	0.00128	3.00E-04	0.004497
Response to elevated platelet cytosolic Ca2+	6	148	0.009469	3.81E-04	0.005333
p130Cas linkage to MAPK signaling for integrins	3	22	0.001408	3.95E-04	0.005535
Plasma lipoprotein remodeling	4	56	0.003583	4.73E-04	0.006149
Plasma lipoprotein assembly, remodeling, and clearance	5	102	0.006526	5.09E-04	0.006617
Platelet activation, signaling and aggregation	8	293	0.018746	5.75E-04	0.006899
ER-Phagosome pathway	5	106	0.006782	6.05E-04	0.007258
Keratinization	7	226	0.014459	6.17E-04	0.007402
MTF1 activates gene expression	2	6	3.84E-04	6.97E-04	0.007671
Binding and Uptake of Ligands by Scavenger Receptors	6	168	0.010749	7.37E-04	0.008102
Assembly of collagen fibrils and other multimeric structures	4	67	0.004287	9.19E-04	0.010111
Collagen degradation	4	69	0.004415	0.001024	0.010492
Initial triggering of complement	5	120	0.007678	0.001049	0.010492
Signaling by PDGF	4	70	0.004479	0.00108	0.010795
Glutamate Neurotransmitter Release Cycle	3	32	0.002047	0.001162	0.011623
Antimicrobial peptides	5	123	0.007869	0.00117	0.011696
Antigen processing-Cross presentation	5	128	0.008189	0.001393	0.012533
Collagen biosynthesis and modifying enzymes	4	76	0.004862	0.001457	0.013116
MyD88:MAL(TIRAP) cascade initiated on plasma membrane	5	133	0.008509	0.001645	0.013164
Toll Like Receptor TLR6:TLR2 Cascade	5	133	0.008509	0.001645	0.013164
Toll Like Receptor TLR1:TLR2 Cascade	5	136	0.008701	0.001813	0.0145
Toll Like Receptor 2 (TLR2) Cascade	5	136	0.008701	0.001813	0.0145
Integrin signaling	3	39	0.002495	0.002038	0.016303
Degradation of the extracellular matrix	5	148	0.009469	0.002607	0.020024
Signaling by high-kinase activity BRAF mutants	3	44	0.002815	0.002861	0.020024
NCAM1 interactions	3	44	0.002815	0.002861	0.020024
Dermatan sulfate biosynthesis	2	13	8.32E-04	0.003181	0.022264
Classical antibody-mediated complement activation	4	97	0.006206	0.003497	0.024478
MAP2K and MAPK activation	3	49	0.003135	0.003862	0.025919
Signaling by RAF1 mutants	3	49	0.003135	0.003862	0.025919
Assembly and cell surface presentation of NMDA receptors	3	49	0.003135	0.003862	0.025919
Anchoring fibril formation	2	15	9.60E-04	0.0042	0.025919
Toll Like Receptor 4 (TLR4) Cascade	5	166	0.010621	0.004235	0.025919
FCGR activation	4	103	0.00659	0.00432	0.025919
Collagen formation	4	104	0.006654	0.004469	0.026812
Gap junction trafficking	3	52	0.003327	0.004553	0.027318
Transport of small molecules	14	1016	0.065003	0.004854	0.029125
Signaling by moderate kinase activity BRAF mutants	3	54	0.003455	0.005053	0.030316
Signaling downstream of RAS mutants	3	54	0.003455	0.005053	0.030316
Paradoxical activation of RAF signaling by kinase inactive BRAF	3	54	0.003455	0.005053	0.030316
Signaling by RAS mutants	3	54	0.003455	0.005053	0.030316
Creation of C4 and C2 activators	4	111	0.007102	0.005608	0.033649
Gap junction trafficking and regulation	3	57	0.003647	0.005862	0.035172
Non-integrin membrane-ECM interactions	3	61	0.003903	0.007056	0.040158
Response to metal ions	2	21	0.001344	0.008032	0.040158

Pathway name	#Entities found	#Entities total	Entities ratio	Entities pValue	Entities FDR
Factors involved in megakaryocyte development and platelet production	5	194	0.012412	0.008045	0.040225
Microtubule-dependent trafficking of connexons from Golgi to the plasma membrane	2	22	0.001408	0.008779	0.043894
Role of phospholipids in phagocytosis	4	129	0.008253	0.009389	0.046946
Kinesins	4	68	0.004351	0.009469	0.047346
Transport of connexons to the plasma membrane	2	23	0.001472	0.009556	0.047779

FDR & *p*-value <0.05

Supplementary table S7: Models' parameters.

Number of Total Samples	Number of GBM Samples	% of Correct Classification Rate
6	3	98.08
8	4	98.15
12	6	97.25
16	8	97.67
24	12	99.54
32	16	99.24
40	20	98.47
48	24	98.25
56	28	98.31
64	32	98.67
76	38	98.77

Supplementary figure S1:

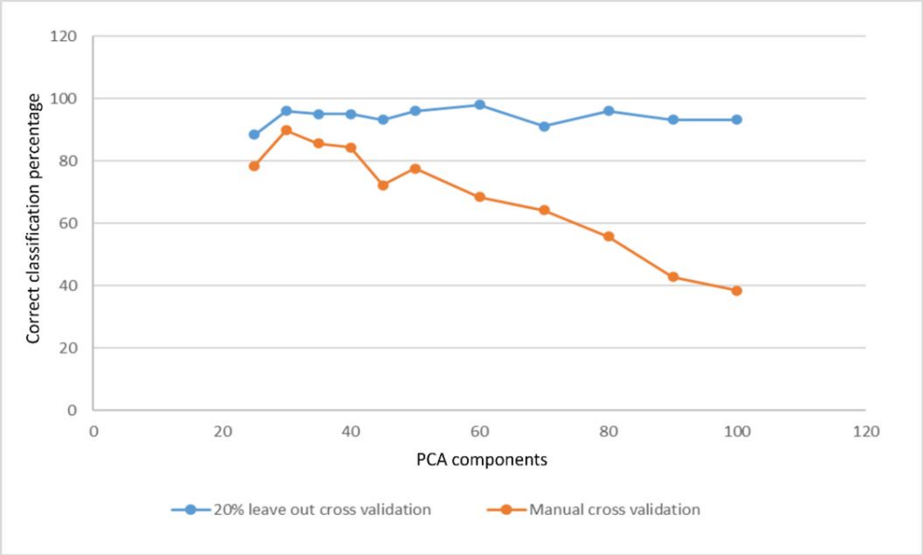

Supplementary figure S1: Line chart illustrating the relationship between the number of PCA components and the correct classification rates of potential models.

Supplementary figure S2:

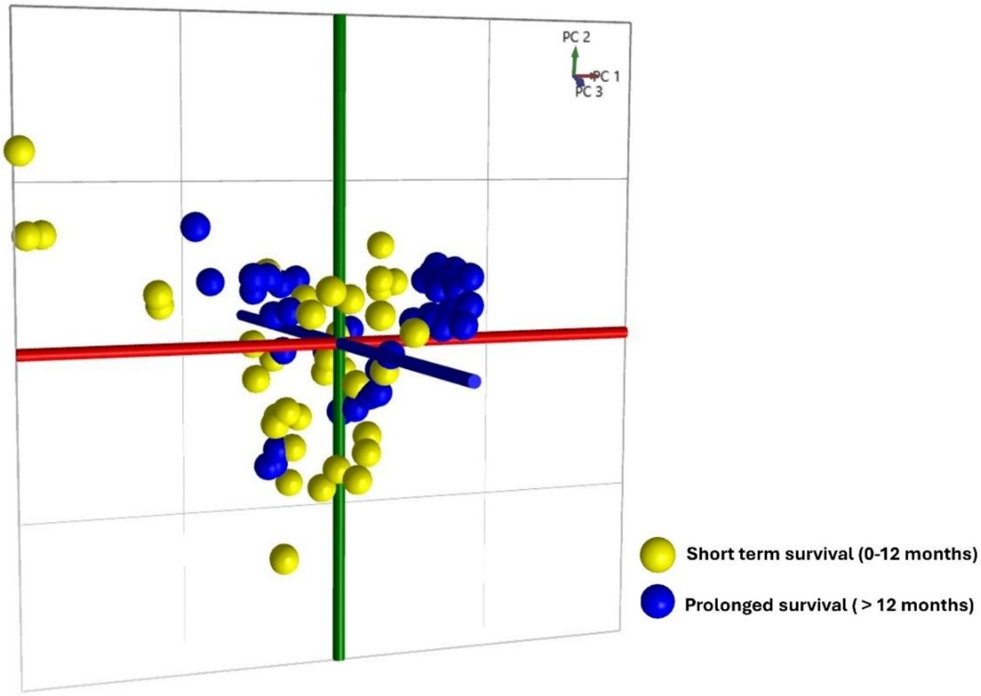

Supplementary figure S2: PCA model classifying human GBM IV patient overall survival times.

Supplementary figure S3:

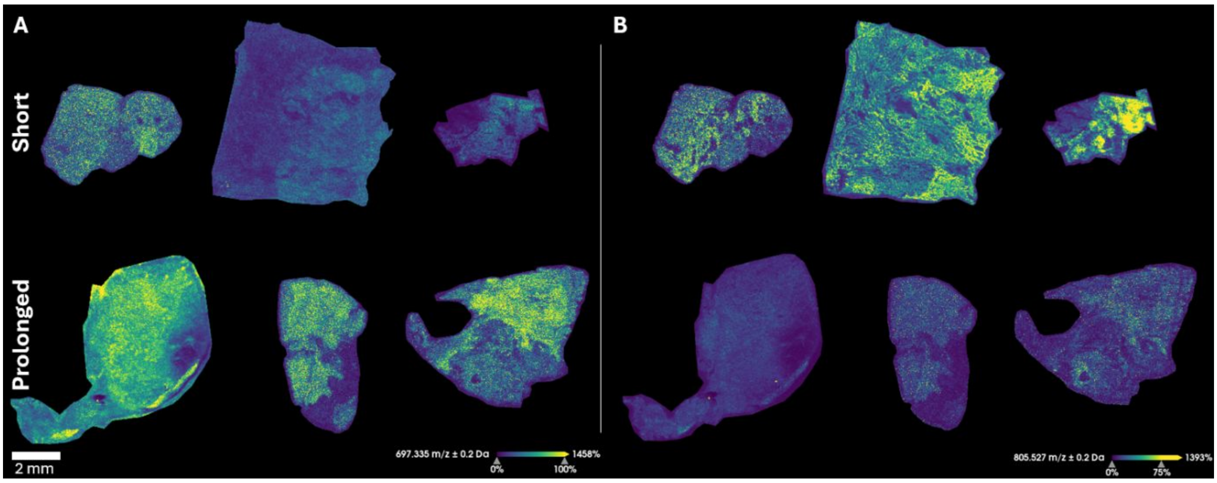

Supplementary figure S3: MALDI-MSI (-) of short- and prolonged survivor glioblastoma sections. Highlighted are m/z 697.335 (A) and PC (38:5)-H (B) normalized by the peak area of m/z 333.147 (norharmane matrix).

Supplementary figure S4:

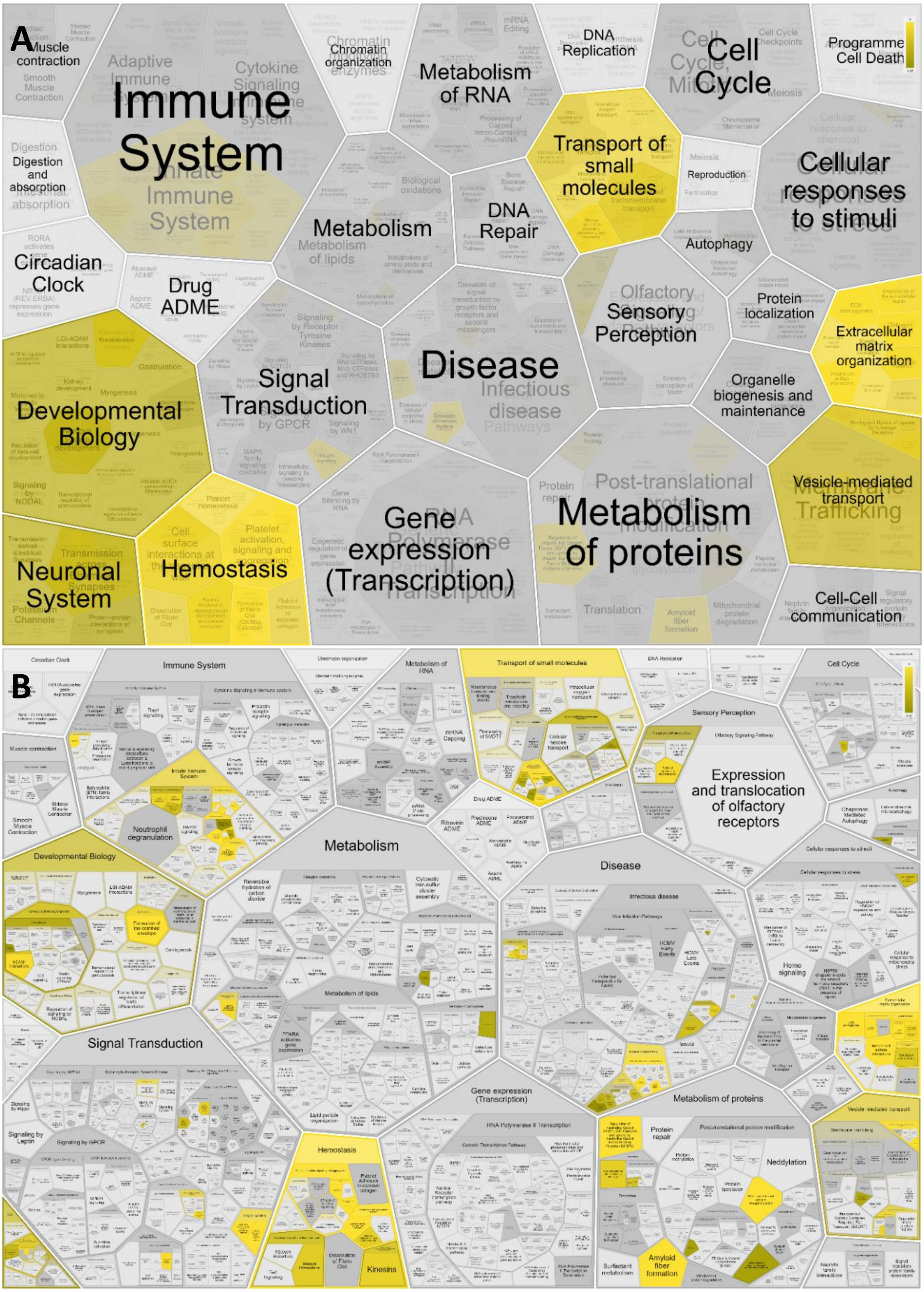

Supplementary figure S4: Reactome overview of significantly altered pathways between short-term and prolonged survivors combined. (A) shows the hierachical view and (B) shows the flattened view.

Supplementary figure S5:

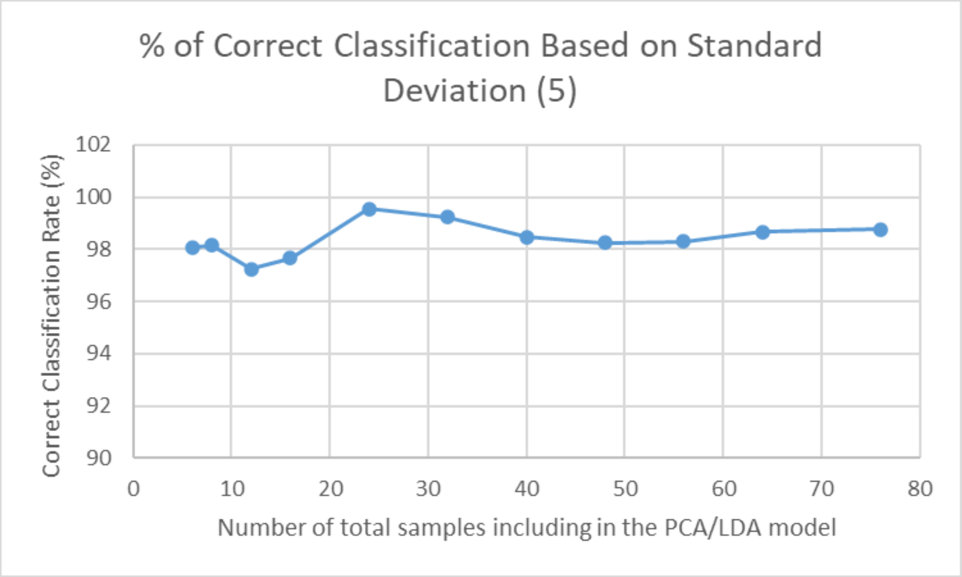

Supplementary figure S5: Line chart illustrating the relationship between the sample size and classification accuracy for different-sized models.
